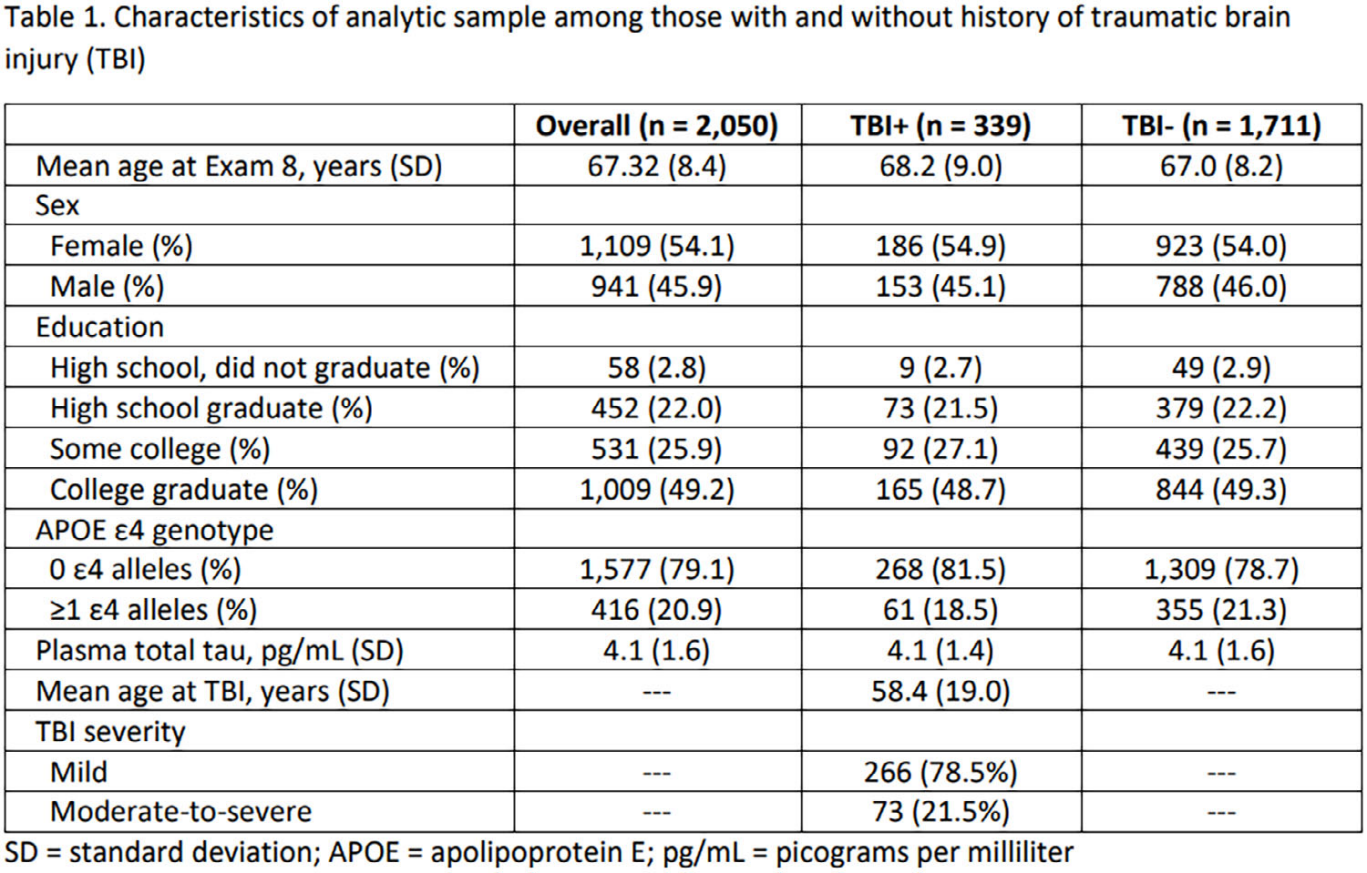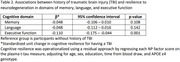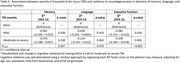# Traumatic brain injury and cognitive resilience in the Framingham Heart Study

**DOI:** 10.1002/alz.088547

**Published:** 2025-01-09

**Authors:** Phillip H Hwang, Shruti Durape, Eden Price, Ashita S. Gurnani, Ting Fang Alvin Ang, Sherral A. Devine, Seo‐Eun Choi, Michael L. Lee, Phoebe Scollard, Laura E. Gibbons, Shubhabrata Mukherjee, Emily H. Trittschuh, Richard Sherva, Logan C. Dumitrescu, Timothy J. Hohman, Andrew J. Saykin, Paul K. Crane, Yorghos Tripodis, Michael L. Alosco, Douglas I Katz, Kristen Dams‐O'Connor, Rhoda Au, Lindsay A. Farrer, Jesse Mez

**Affiliations:** ^1^ The Framingham Heart Study, Framingham, MA USA; ^2^ Boston University School of Public Heatlh, Boston, MA USA; ^3^ Department of Neurology, Boston University Chobanian & Avedisian School of Medicine, Boston, MA USA; ^4^ Framingham Heart Study, Boston University Chobanian & Avedisian School of Medicine, Boston, MA USA; ^5^ Boston University Alzheimer’s Disease Research and CTE Centers, Boston University Chobanian & Avedisian School of Medicine, Boston, MA USA; ^6^ Framigham Heart Study ‐ Boston University, Framingham, MA USA; ^7^ Boston University Chobanian & Avedisian School of Medicine, Boston, MA USA; ^8^ Framingham Heart Study, Framingham, MA USA; ^9^ Slone Epidemiology Center, Boston University Chobanian & Avedisian School of Medicine, Boston, MA USA; ^10^ Department of Anatomy & Neurobiology, Boston University Chobanian & Avedisian School of Medicine, Boston, MA USA; ^11^ Department of General Internal Medicine, University of Washington School of Medicine, Seattle, WA USA; ^12^ Department of General Internal Medicine, University of Washington, Seattle, WA USA; ^13^ University of Washington Alzheimer’s Disease Research Center, University of Washington School of Medicine, Seattle, WA USA; ^14^ University of Washington, Seattle, WA USA; ^15^ University of Washington, School of Medicine, Seattle, WA USA; ^16^ Geriatric Research, Education, and Clinical Center, Veterans Affairs Puget Sound Health Care System, Seattle, WA USA; ^17^ Department of Medicine (Biomedical Genetics), Boston University Chobanian & Avedisian School of Medicine, Boston, MA USA; ^18^ Vanderbilt Memory and Alzheimer’s Center, Vanderbilt University Medical Center, Nashville, TN USA; ^19^ Vanderbilt Genetics Institute, Vanderbilt University Medical Center, Nashville, TN USA; ^20^ Vanderbilt Memory & Alzheimer’s Center, Department of Neurology, Vanderbilt University Medical Center, Nashville, TN USA; ^21^ Department of Pharmacology, Vanderbilt University, Nashville, TN USA; ^22^ Department of Neurology, Vanderbilt University Medical Center, Nashville, TN USA; ^23^ Indiana Alzheimer’s Disease Research Center, Indianapolis, IN USA; ^24^ Department of Neurology, Indiana University School of Medicine, Indianapolis, IN USA; ^25^ Department of Medical and Molecular Genetics, Indiana University School of Medicine, Indianapolis, IN USA; ^26^ Indiana Alzheimer’s Disease Research Center, Indiana University School of Medicine, Indianapolis, IN USA; ^27^ Stark Neurosciences Research Institute, Indiana University School of Medicine, Indianapolis, IN USA; ^28^ University of Washington Alzheimer's Disease Research Center, University of Washington School of Medicine, Seattle, WA USA; ^29^ Boston University Alzheimer’s Disease Research Center, Boston, MA USA; ^30^ Boston University School of Public Health, Boston, MA USA; ^31^ Icahn School of Medicine at Mount Sinai, New York, NY USA; ^32^ Department of Epidemiology, Boston University School of Public Health, Boston, MA USA; ^33^ Department of Anatomy and Neurobiology, Neurology and Medicine, Framingham Heart Study, BU Alzheimer’s Disease Research Center, Boston University Chobanian & Avedisian School of Medicine, Boston, MA USA; ^34^ Alzheimer’s Disease Research Center, Boston University Chobanian & Avedisian School of Medicine, Boston, MA USA; ^35^ Departments of Medicine (Biomedical Genetics), Neurology, Ophthalmology, Biostatistics, and Epidemiology, Boston University Schools of Medicine and Public Health, Boston, MA USA; ^36^ Department of Biostatistics, Boston University School of Public Health, Boston, MA USA; ^37^ Framingham Heart Study, Boston, MA USA

## Abstract

**Background:**

Some evidence supports an association between traumatic brain injury (TBI) and greater risk of dementia, but the role of cognitive resilience in this association is poorly understood.

**Method:**

2,050 participants from the Framingham Heart Study Offspring cohort who were aged ≥60 year and had a plasma total tau (t‐tau) measure at Exam 8 (2005‐2008), and a neuropsychological (NP) exam visit within five years were included. Plasma t‐tau was measured using the Simoa assay (Quanterix). NP factor scores were previously derived for memory, language, and executive function using confirmatory factor analysis. Information on TBIs was collected by comprehensive review of medical records, health history updates, exams, and self‐report. TBI occurrence and severity were operationalized using modified ACRM & VA/DoD criteria, respectively. Cognitive resilience was operationalized using a residual approach by regressing each NP factor score on the plasma t‐tau measure, adjusting for age at Exam 8, sex, education, time from blood draw, and *APOE* ε4 genotype. The adjusted residuals were then regressed on history of TBI (yes versus no), and severity of TBI (moderate‐to‐severe versus mild versus none).

**Result:**

The sample was, on average, 67 years of age at Exam 8, 54% female, and college educated. No differences were observed in plasma t‐tau levels between those with and without TBI. Having a history of TBI was significantly associated with a reduction in resilience in executive function (β: ‐0.110; 95% CI: ‐0.175, ‐0.044; p: 0.001) as compared to not having a history of TBI. No significant associations were observed between history of TBI and resilience in memory or language. Greater TBI severity was significantly associated with worse resilience in executive function in a dose‐response manner (P_trend_: <0.001), with the association being strongest in the moderate‐to‐severe TBI group (β: ‐0.209; 95% CI: ‐0.340, ‐0.078; p: 0.002) followed by the mild TBI group (β: ‐0.082; 95% CI: ‐0.155, ‐0.010; p: 0.026).

**Conclusion:**

Having a TBI was associated with worse resilience to neurodegeneration in executive function, and most strongly among individuals with moderate‐to‐severe TBI. These results suggest that having a TBI may increase vulnerability to late‐life executive dysfunction after accounting for a primary neurodegenerative disease process.